# Long-Term Outcome after Cholecystectomy without Common Bile Duct Catheterization and Flushing in Dogs

**DOI:** 10.3390/ani12162112

**Published:** 2022-08-17

**Authors:** Matteo Rossanese, Phillipa Williams, Andrew Tomlinson, Filippo Cinti

**Affiliations:** 1 Department of Clinical Science and Services, Royal Veterinary College, London NW1 0TU, UK; 2 Dick White Referrals, Six Mile Bottom CB8 0UH, UK; 3 Small Animal Teaching Hospital, University of Liverpool, Neston CH64 7TE, UK; 4 Ospedale Veterinario I Portoni Rossi-Anicura, 40069 Bologna, Italy

**Keywords:** gall bladder mucocele, common bile duct, laparoscopy, cholecystectomy, dogs

## Abstract

**Simple Summary:**

Gall bladder mucocele (GBM) is one of the most common indications for biliary surgery in dogs, and cholecystectomy is considered the treatment of choice. Before cystic duct ligation, catheterization and flushing of the common bile duct (CBD) is commonly performed to ensure patency but is potentially associated with complications. Laparoscopic cholecystectomy (LC) has been described in dogs as a treatment for uncomplicated GBM without evidence of biliary tract rupture or CBD obstruction. In this study, we describe the long-term outcome of dogs undergoing cholecystectomy for GBM without catheterization and flushing of the CBD. The results of the present study suggest that this cohort of dogs had a good-to-excellent long-term prognosis, with a low morbidity and perioperative mortality, with 96% of dogs surviving for more than 6 months. This population could represent ideal candidates for LC in dogs.

**Abstract:**

The aim of this study is to report outcomes of dogs undergoing cholecystectomy for gall bladder mucocele (GBM) without flushing and catheterization of the common bile duct (CBD). This is a retrospective multicentric study from three veterinary referral hospitals and included 82 dogs diagnosed with GBM. Medical records were reviewed for clinical and histopathological findings. Long-term outcome was assessed with an owner questionnaire. The common bile duct was considered normal (<4 mm), mildly dilated (5–6 mm) and moderately dilated (>7 mm) in 88%, 10% and 2.4% of dogs, respectively. Surgery was uncomplicated in 83% of dogs. Intraoperative complications were recorded in 21% of dogs, with hypotension being the most common, whereas postoperative complications were documented in 20% of dogs, with vomiting/regurgitation being the most common. Ninety-six percent of dogs that underwent cholecystectomy in this study survived to discharge. Follow-up ranged from 142 to 3930 days (median: 549 days). Eighty-five percent of dogs were alive at the time of follow-up. Dogs undergoing cholecystectomy for GBM without catheterization and flushing of the CBD have a favourable prognosis for recovery and quality of life.

## 1. Introduction

Gall bladder mucocele (GBM) resulting from excessive mucin secretion and production of hyperviscous bile is one of the most common indications for biliary surgery in dogs [1,2,3,4,5,6,7,8,9,10]. Migration of bile-laden mucus into the cystic, hepatic, and common bile ducts may result in various degrees of extrahepatic biliary obstruction (EHBO) [1,5,6,11,12]. The etiology of GBM remains unclear, although it is thought to be multifactorial [1,2]. Cholecystectomy is considered the treatment of choice for dogs with GBM and often involves catheterization and flushing of the common bile duct (CBD) to ensure patency before cystic duct ligation [1,2,5,6,13,14,15]. Common bile duct flushing can be undertaken either by normograde catheterization via cholecystotomy, before cholecystectomy, or retrograde catheterization via the major duodenal papilla after duodenotomy [1,2,3,5,7,9,10,12,13,14,16,17]. The proposed benefit of CBD flushing is to reduce the risk of postoperative compromised bile flow due to residual bile plugs or choleliths [2,3,6,9,10,12,17]. However, the absolute requirement for intraoperative CBD catheterization remains controversial. Pike et al. (2004) reported that dogs with high preoperative serum bilirubin concentrations and intraoperative gross distension of the CBD that underwent cholecystectomy without CBD catheterization and flushing experienced excellent clinical outcomes, suggesting that residual mucous within the biliary tree passed after the gall bladder was removed [1].

Laparoscopic cholecystectomy (LC) is considered the gold-standard treatment for gall bladder removal in humans [18]. This minimally invasive approach has been described in dogs as treatment for uncomplicated GBM without evidence of biliary tract rupture or obstruction [17,18,19,20,21,22]. During LC, the patency of the CBD cannot be easily assessed; therefore, appropriate case selection in dogs is important before undertaking this minimally invasive approach. In humans, absolute contraindications for laparoscopic cholecystectomy are the inability of a patient to tolerate carbon dioxide pneumoperitoneum and diffuse peritonitis with hemodynamic compromise [18]. Relative contraindications include gall bladder cancer, cirrhosis with portal hypertension, severe cardiopulmonary disease, bleeding disorders and multiple prior operations [18]. Criteria for case selection for LC in dogs have previously been described as no evidence of biliary obstruction either on abdominal ultrasound examination or demonstrated on serum total bilirubin concentration [17]. However, Scott et al. (2016) suggested that dogs with mild elevations in serum total bilirubin concentrations may be acceptable candidates for LC [22]. Factors that may indicate the need for conversion to an open cholecystectomy have not been identified [22]. Perioperative complications [22] and short-term outcomes [20,21] have been described; however, there is limited long-term data on dogs undergoing cholecystectomy without CBD flushing. The aim of this study was to describe long-term outcomes of dogs undergoing traditional open cholecystectomy for GBM without catheterization and flushing of the CBD. We hypothesized that the outcome for this population would be good to excellent and that these dogs could represent ideal candidates for LC.

## 2. Materials and Methods

### Study Design and Eligibility Criteria

This retrospective cohort observational study used anonymized clinical data and was approved by the Royal College Research Ethics Committee (URN SR2020-0313) and the University of Liverpool Ethics Committee (VREC1080). Medical records from three small animal referral institutions were searched to identify all dogs that underwent cholecystectomy between 2014 and 2021.

Inclusion criteria for the study required comprehensive clinical records, a detailed surgical report stating that no intervention to the biliary system was performed during the cholecystectomy procedure and a follow-up period of at least 6 months.

Dogs were excluded from the study if catheterization and flushing of the CBD were performed during the surgical procedure either normograde, through a cholecystotomy, or retrograde, through a duodenotomy. Dogs that had undergone previous surgery involving the biliary tract or those that had evidence of gall bladder or biliary tract rupture were also excluded.

Information retrieved from the records included signalment (age, body weight, body condition score, breed, gender and neuter status), clinical history and presenting clinical signs, examination findings, preoperative blood test results, preoperative diagnostic investigations and findings (radiography, computed tomography, magnetic resonance imaging, ultrasonography and echocardiography), time from presentation to surgery (days), surgical treatment and concomitant surgical procedures under the same general anaesthetic, histopathological findings, time from surgery to discharge (days), bacteriology results, and survival to hospital discharge. The etiological cause leading to cholecystectomy was determined on the basis of diagnostic and histopathological findings. The CBD was considered dilated if >4 mm in diameter and classified as mildly dilated (5–6 mm), moderately dilated (7–8 mm) or severely dilated (>8 mm). The occurrence of any intraoperative and postoperative complications was recorded, as well as the need for further surgical intervention or medical treatment. To investigate any unreported complications, the referring veterinarians and the owners were contacted, and the clinical notes of each patient were reviewed. Follow-up phone conversations and/or clinical examination with owners determined outcomes. If the dog had died or was euthanized, the cause of death was recorded. Descriptive statistics were computed for all variables. Analyses were performed using Microsoft Excel 2020 and SPSS 26.0 (IBM SPSS statistics, version 26.0; IBM Corp, Armonk, NY, USA). The Shapiro—Wilk test was used to confirm that none of the assessed continuous variables were normally distributed (*p* < 0.001 for all), so they are reported as median (range). Statistical analysis was not performed due to the small population size.

## 3. Results

### 3.1. Population Data, Clinical Presentation and Diagnostic Investigations

In total, 82 dogs met the eligibility criteria. The most represented breeds were border terrier (32%), miniature schnauzer (8.5%), crossbreed (8.5%), cocker spaniel (6%), Bichon Frisé (6%), Jack Russell terrier (6%), chihuahua (5%), Yorkshire terrier (3.6%), Labrador retriever (3.6%), beagle (3.6%) and others.

The population included 41 male dogs (32 neutered and 9 intact) and 41 female dogs (38 neutered and 3 intact). At the time of surgery, the median age was 108 months (range, 48 to 168), and the median weight was 10 kg (range, 3.7 to 31.8). The median body condition score was 5/9 (range, 3/9 to 9/9).

Median duration of clinical signs was 7 days, ranging from less than 24 h prior to presentation to 120 days. 

The most common clinical sign was vomiting (79%), followed by lethargy (56%), anorexia/hyporexia (39%), diarrhoea (22%), jaundice (10%) polyuria/polydipsia (10%) and abdominal pain (7%). In the majority of dogs, a combination of clinical signs was reported.

Findings upon physical examination included abdominal pain (48%), icterus (10%), pyrexia (5%), altered mental status (2%), 5% dehydration (2%) and distended abdomen (2%). In 37 dogs (45%), physical examination revealed no abnormalities.

Complete blood count (CBC) was available in 78 dogs, and abnormalities included neutrophilia (>12 × 10^9^/L) in 28 dogs (36%), with band neutrophils in 8 dogs, leukocytosis (>18 × 10^9^/L) in 21 dogs (27%) and anaemia (HCT < 35%) in 6 dogs (8%). In 57 dogs (72%), CBC was within reference limits.

Serum biochemistry was available in all dogs (Table 1). Hypoproteinaemia and hypoalbuminaemia were present in 6 dogs (7%) and 10 dogs (12%), respectively. Alkaline phosphatase levels (ALP) were increased (>212 U/L) in 68 (83%) dogs, and alanine aminotransferase levels were increased (>125 U/L) in 51 dogs (62%). Hypercholesterolaemia (cholesterol > 8.3 mmol/L) was present in 39 dogs (48%), and hyperbilirubinaemia (total bilirubin >15 μmol/L) was present in 30 dogs (37%). In 12 dogs (15%), serum biochemistry was within normal limits.

Concomitant endocrine diseases were present in 21 dogs (26%); eleven dogs (13%) were diagnosed with hyperadrenocorticism, nine dogs (11%) with diabetes mellitus and one dog with exocrine pancreatic insufficiency (1.2%).

Coagulation parameters (prothrombin time and partial thromboplastin time) were assessed in 42 dogs and were within reference intervals in 38 dogs (90%).

**Figure 1:**
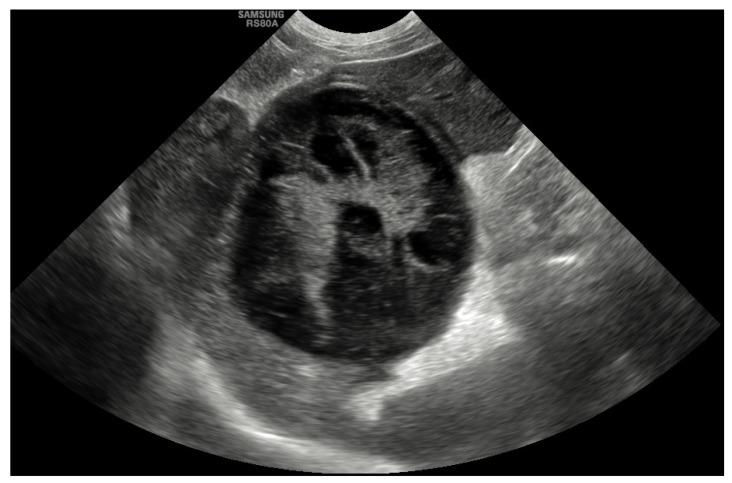
Sagittal ultrasonographic image of a gall bladder mucocele with multiple hyperechoic areas with a stellate appearance in an 8-year-old female neutered border terrier.

Abdominal ultrasound was the most common diagnostic tool used to diagnose GBM and was performed in all cases (100%). The most common abnormality that was described in 45 dogs (55%) was evidence of a GBM with a stellate or so-called “kiwi-fruit” pattern (Figure 1). A combination of a distended and thick gallbladder with hyperechoic and organized bile sediment compatible with an early GBM was described in the other 37 cases (45%). Hyperechoic pericholecystic fat was described in 11 dogs, and free peritoneal fluid was reported in 7 dogs. Other findings included hepatomegaly (15%), congenital extrahepatic portosystemic shunt (EHPSS; 2.5%), liver nodules (7%), splenic nodules (1.2%), bilateral adrenal gland enlargement (1.2%) and bladder nodules (1.2%). The common bile duct was considered to be within the normal limits (<4 mm) in 72 dogs (88%), mildly dilated (5–6 mm) in 8 dogs (10%) and moderately dilated (>7 mm) in 2 dogs (2.4%). The intrahepatic ducts were considered normal in all cases.

Other investigations included radiographs, which were performed in 29 cases (35%); echocardiography in 4 cases (5%); and computed tomography and MRI in 3 (4%) cases, respectively.

### 3.2. Surgical Procedures

The median duration between the diagnosis of gallbladder disease and surgery was 4 days (range, 1–180).

All surgeries were performed by or under the direct supervision of a European College of Veterinary Surgeons (ECVS) board-certified or Royal College of Veterinary Surgeons (RCVS) specialist surgeon. Surgery included cholecystectomy without either normograde or retrograde catheterisation and flushing of the CBD. Subjectively, mild-to-moderate dilation of the CBD was reported in 12 dogs, without evidence of obstruction. 

A total of 73 concomitant surgeries were performed in 63 dogs, including liver biopsies (74%), oesophagostomy tube placement (7%), gastrointestinal biopsies (5%), pancreatic biopsies (1.2%) and urinary bladder biopsies (1.2%). Abdominal lavage was performed using warm isotonic sterile saline in 43 dogs (52%). Postoperative hospitalization for monitoring and analgesia ranged from 1 to 6 days (median: 3 days). Antibacterial and analgesia therapy was prescribed postoperatively at the discretion of the surgeon; a total of 37 dogs (45%) received prophylactic antibiotics postoperatively. 

### 3.3. Clinicopathological Results

A gall bladder mucocele was confirmed in all dogs upon histopathological analysis. Associated mucinous cystic hyperplasia was reported in 22 dogs (27%), lymphoplasmacytic and neutrophilic cholecystitis in 18 dogs (22%), chronic lymphoplasmacytic cholecystitis in 11 dogs (13%), necrosuppurative cholecystitis in 9 dogs (11%) and gallbladder adenoma in 1 dog (1%).

Aerobic and anaerobic bacterial culture and antimicrobial susceptibility testing from a bile sample or gallbladder wall sample were performed in 80 dogs (98%). In 16 cases (20%), bacterial culture was positive, including mixed growth of *Escherichia coli* (50%), *Enterococcus* spp. (30%), *Staphylococcus* spp. (5%)*, Proteus* spp. (5%) and *Clostridium perfringens* (5%).

### 3.4. Complications

Surgery was uncomplicated in 68 dogs. Seventeen dogs (21%) had at least one intraoperative complication recorded. Hypotension was documented in 14 dogs (17); 3 dogs (4%) suffered mild bleeding from the hepatic fossa, and one suffered moderate bleeding, requiring blood-product transfusion. Three dogs (4%) exhibited intraoperative regurgitation, and one of these dogs was diagnosed with aspiration pneumonia postoperatively. During hospitalization, 17 complications occurred in 16 dogs (20%), which included vomiting/regurgitation (65%), pancreatitis (23%), hemoabdomen requiring blood-product transfusion (6%) and urinary tract infection (6%). All dogs survived the surgical procedure, but three dogs (3.7%) died prior to discharge. One dog that had a blood-product transfusion due to ongoing bleeding, suffered another severe episode of hypotension and was euthanized. One dog suffered cardiorespiratory arrest and died. One dog developed an acute kidney injury and was euthanized.

### 3.5. Outcomes

A total of 79/82 (96%) dogs that underwent cholecystectomy without CBD catheterization and flushing survived to discharge.

Follow-up was available for all dogs and ranged from 142 to 3930 days (median: 549 days). Sixty-seven dogs (85%) were alive at the time of follow-up. Twelve dogs (15%) died or were euthanized during the follow-up period of between 142 and 2910 days, and nine dogs (11%) died for reasons unrelated to the cause of the GBM.

Three dogs (4%) died or were euthanized for causes potentially related to the cause of the GBM between 142 and 366 days, including signs related to the gastrointestinal system, vomiting, collapse and pancreatitis.

Median time to repeat postoperative hematology and serum biochemistry was 30 days (range, 14–270). All biochemistry parameters improved during the postoperative period (Table 1).

During the follow up period, 26 dogs (39%) had a postoperative abdominal ultrasound, and mild-to-moderate CBD dilation (5–8 mm) was reported in all of them without evidence of biliary outflow obstruction. Intrahepatic cholelithiasis and mild dilation of the intrahepatic biliary duct were reported in one dog. Long-term issues were reported in only one dog; CBD dilation with hyperechoic material was documented 11 months after cholecystectomy. Repeated surgery revealed a CBD markedly distended with inspissated bile and choleliths. Choledochotomy was performed, and the CBD was catheterized and flushed. The dog recovered well from the surgery, and no further issues were reported. Fifty-one owners (76%) of the surviving dogs were contacted. All owners considered the results of surgery to be excellent, with complete resolution of preoperative clinical signs. The quality of life of the dogs was described as excellent in 45 cases (88%) and good in 6 cases (12%).

## 4. Discussion

In this study, dogs that underwent cholecystectomy for GBM without catheterization and flushing of the CBD had a good-to-excellent long-term prognosis, with a low morbidity and perioperative mortality, leading us to accept our hypothesis.

Historically, cholecystectomy has been associated with a mortality rate as high as 40%; however, the prognosis improves if the patient survives the immediate postoperative period [1,3,5,6,15,23,24]. Youn et al. (2018) reported an overall mortality rate of 9%, but when considering only elective cholecystectomies, the mortality rate was only 2% [15]. In this described cohort of dogs, 58% did not have aspiration or catheterization of the CBD [15], and no dogs required additional surgery for biliary obstruction during the hospitalization period. However, no information regarding the long-term outcomes was available [15].

In the current study, the overall perioperative mortality rate was 4%; this was comparable to other recent studies, reflecting the current trend toward earlier surgical treatment of dogs in which GBM has been diagnosed [8,9,10,15].

In our experience, CBD flushing is performed if there is evidence of a clear dilation of CBD and intrahepatic ducts on preoperative imaging causing a bile outflow obstruction, with the final decision taken intraoperatively with a direct visualization of the CBD. Catheterization and flushing of the CBD is not performed in dogs with absent-to-minimal dilation of the CBD.

A previous study failed to report any significant association with survival for dogs undergoing open cholecystectomy for GBM where the common bile duct was catheterized to ensure patency, compared to those dogs in which common bile duct patency was not assessed [6].

Piegols et al. (2020) reported that catheterization of the CBD was associated with an increased surgical time and an increased risk of pancreatitis, with no differences recorded between normograde catheterization through cholecystotomy and antegrade catheterization via duodenotomy. Moreover, it was not associated with a more rapid rate of decline of serum bilirubin levels postoperatively [9,10]. Similarly, Putterman et al. (2021) reported that dogs catheterized retrograde were more likely to experience postoperative complications, including persistence of gastrointestinal signs, when compared to normograde catheterization [10].

It is possible that flushing of the CBD causes reflux of biliary secretions into the pancreatic duct, resulting in pancreatitis, or that the placement of a catheter may result in trauma to the major duodenal papilla, resulting in inflammation and transient occlusion of the pancreatic duct orifice at this site [9,10]

In this study, the only confirmed long-term complication associated with the CBD was reported in one dog that re-presented with a severe CBD dilation and recurrence of biliary obstruction due to inspissated bile and choleliths. It is unknown whether this complication could have been avoided if catheterization and flushing of the CBD were performed during the initial surgery. In this case, the choleliths were subsequently removed through a choledochotomy, and the CBD was catheterized and flushed to ensure patency.

Laparoscopic cholecystectomy is a well-established procedure in human medicine and is increasingly advocated for use in dogs [17,19,20,21,22].

The use of LC has been reported in dogs for benign gallbladder diseases, such as gallbladder mucocele, cholelithiasis and cholangiohepatitis or for dogs that do not have evidence of extrahepatic biliary obstruction [17,19,22]. Assessment of extrahepatic biliary obstruction under laparoscopy is difficult in dogs, and confirmation of patency of the CBD is not commonly reported [20,21,22]. In human medicine, this is performed, if necessary, using intraoperative cholangiogram under laparoscopic and fluoroscopic guidance [18,20,21]. Indications include an unclear biliary anatomy, a concern for choledocholithiasis or a concern for bile duct injury [18].

Hayakawa et al. (2018) described that CT cholangiography revealed the structural characteristics of the canine bile duct system with minimal invasiveness, showing the passage of the contrast medium into the duodenum. This technique could demonstrate EHBO and could be used as preoperative evaluation to ensure CBD patency [16].

Intraoperative CBD flushing and cholangiography was reported in two recent veterinary studies; this was performed only in selected cases when CBD obstruction was suspected, and it was described as a useful tool to detect obstructions and stricture in the CBD [20,21,22].

In veterinary medicine, there are no clear guidelines to identify candidates for LC; current recommendations are based on a subjective interpretation of imaging and laboratory test results [17]. According to Mayhew et al. (2008), good candidates for LC are dogs with GBM that are not associated with biliary tract rupture or obstruction upon abdominal ultrasound examination or serum total bilirubin concentration [17]. Scott et al. (2016) found no differences in the serum total bilirubin concentration for dogs undergoing LC and dogs requiring conversion to open cholecystectomy [22]. Multiple studies failed to find significant differences in serum total bilirubin concentration, serum alkaline phosphatase, alanine transaminase and gamma-glutamyl transferase activities in dogs undergoing open cholecystectomy, regardless of the outcome [6,7,8,9,10,11,12,13,14,15,16,17,18,19,20,21,22].

In this population, the preoperative serum total bilirubin level ranged from 2.2 to 204 mmol/L, whereas the postoperative serum total bilirubin level ranged from 1.2 to 73 mmol/L, with an effective median decrease of 47% post-surgery.

The findings of previous studies, along with the results of this study, suggest that dogs with elevations in serum total bilirubin concentrations do not necessarily require catheterization and flushing of the CBD, and they may be acceptable candidates for LC [6,7,8,9,10,11,12,13,14,15,16,17,18,19,20,21,22].

In this study, postoperative ultrasonographic assessment revealed a mild-to-moderate dilation of the CBD (ranging from 5 to 8 mm) in dogs with a CBD within the normal limits or only mildly dilated before surgery. This level of dilation without evidence of biliary outflow obstruction is not surprising, and it should not be considered as pathological. Following a hypothesis suggested by Oddi in 1887, many studies reported that the physiological dilatation of the bile duct after cholecystectomy was due to the disappearance of the gallbladder’s reservoir function. This dilatation is considered to be purely compensatory and starts in the early postoperative period until the CBD adapts to contain bile equal to the gallbladder [25,26,27,28].

This study provides evidence that the long-term prognosis following cholecystectomy with CBD catheterization and flushing can be good to excellent, with 96% of dogs surviving for more than 6 months. This is the first study to include owner assessed outcomes, highlighting the fact that these dogs can have an excellent long-term prognosis following cholecystectomy without catheterization and flushing of the CBD. Limitations of this study include its retrospective nature, lack of standardization or procedural protocols and the occasional incomplete medical records. Another limitation is the lack of a control group; despite the favorable prognosis of this cohort of dogs, it is unknown whether CDB catheterization and flushing would have provided further benefit. 

The outcome measures were based on a subjective non-validated questionnaire, which could have led to an incorrect owner perception. The long follow-up period could have resulted in recall bias, resulting in less reliable owner assessments. Additional prospective studies are warranted to identify possible risk factors associated with complications and survival, ideally using a large-scale multicenter prospective design.

## 5. Conclusions 

In conclusion, dogs undergoing cholecystectomy for GBM without catheterization and flushing of the CBD have a favorable prognosis for recovery and quality of life. The dogs presented in this study could represent ideal candidates for LC.

## Figures and Tables

**Table 1:** Comparison of average preoperative and postoperative haematological and biochemical parameters in dogs that underwent cholecystectomy due to a gall bladder mucocele.

	Preoperative		Postoperative		
Blood Parameter	Value (Median)	Range	Value (Median)	Range	Reference Range
Haematology					
RBC	6.2	4.4–7.8	6.9	3.9–8.2	5.5–8.5 × 10^12^/L
HGB	16	9.3–19.7	16.4	9.7–22.2	12–18 g/dL
PCV	43.5	27–53	41.3	31–51	37–55%
WBC	18.5	6.1–38.3	12.7	9.3–22	6.0–15.9 × 10^9^/L
Neutrophils	9.4	3.1–34.5	5.2	2.7–24	3–11 × 10^9^/L
Serum biochemistry					
Total protein	63	33–73	52	31–52	54.9–75.3 g/L
Albumin	27.7	21–41	23	19–31	25–40 g/L
Total bilirubin	19	2.2–204	10	1.2–73	0–16 μmol/L
Alanine transferase	207.5	25–2513	181	52–1731	13–88 IU/L
Alkaline phosphatase	821	44–7694	303	95–4010	14–105 IU/L
Cholesterol	7.6	2.7–21	6	4.4–11.6	3.2–6.2 mmol/L
Urea	5.2	2.9–17.3	5.5	2.3–10.6	2.5–7.4 mmol/L
Creatinine	61.5	21–259	54	36–92	40–145 μmol/L
Coagulation					
PT	7.55	6–17			7.5–9.9 s
APTT	14.3	8–29.5			11–21 s

Abbreviations: ALP, alkaline phosphatase; ALT, alanine aminotransferase; APTT, activated partial thromboplastin time; GGT, gamma-glutamyl transferase; HGB, haemoglobin; PCV, packed cell volume; PT, prothrombin time; RBC, red blood cell count; sec, seconds; WBC, white blood cell count.

## Data Availability

Not applicable.
